# The experience of musculoskeletal symptoms among patients with breast cancer during aromatase inhibitor therapy: A qualitative study

**DOI:** 10.1016/j.apjon.2025.100784

**Published:** 2025-09-13

**Authors:** Yuling Cao, Feng Jing, Lingyun Jiang, Maoting Tian, Jiajia Qiu, Lichen Tang, Yan Hu

**Affiliations:** a School of Nursing, Fudan University, Shanghai, China; b Fudan University Shanghai Cancer Center, Shanghai, China

**Keywords:** Breast cancer, Aromatase inhibitor therapy, Musculoskeletal symptoms, Qualitative study, Symptom experience, Symptom management

## Abstract

**Objective:**

To examine the musculoskeletal symptom experiences of Chinese patients with breast cancer receiving aromatase inhibitor (AI) therapy and identify patient-centered strategies for symptom management.

**Methods:**

A descriptive qualitative study was conducted from June to August 2023 at a tertiary hospital in Shanghai, China. Using purposive sampling with maximum variation, 37 women with hormone receptor–positive (HR+) breast cancer (stages I–III) undergoing AI therapy participated in semi-structured telephone interviews. Data were audio-recorded, transcribed verbatim, and analyzed thematically.

**Results:**

Four themes were identified: (1) Symptom burden—persistent joint pain, stiffness, and fatigue interfered with daily activities; (2) Emotional impact—symptoms provoked anxiety, frustration, and reduced well-being; (3) Social challenges—decreased participation in social and family life led to feelings of isolation; and (4) Coping strategies—pharmacological measures (e.g., calcium and vitamin D supplementation) and non-pharmacological interventions (e.g., exercise, acupuncture) were adopted, though access to reliable information and professional guidance was often inadequate.

**Conclusions:**

Musculoskeletal symptoms related to AI therapy impose considerable physical, emotional, and social burdens on Chinese patients with breast cancer, potentially undermining treatment adherence. Culturally appropriate, patient-centered management incorporating both pharmacological and non-pharmacological interventions is essential to optimize symptom control and quality of life.

## Introduction

Breast cancer is the most frequently diagnosed malignancy among women worldwide, with hormone receptor-positive (HR+) subtypes being the most prevalent category.^1^ Third-generation aromatase inhibitors (AIs) have become the cornerstone of endocrine therapy for postmenopausal HR+ ​patients with breast cancer,^2^ demonstrating proven efficacy in suppressing estrogen production and reducing recurrence risk.^3^ Despite their clinical benefits, AIs are associated with a spectrum of adverse effects, particularly AI-associated musculoskeletal symptoms (AIMSS).^4^ These symptoms, including joint pain, stiffness, and myalgias, affect up to 70% of patients^5^ and often result in treatment discontinuation, which undermines therapeutic outcomes.^6^^,^^7^ The impact of AIMSS extends beyond physical discomfort, exerting profound disruptions on patients' daily functioning, social roles, and emotional well-being.^8^ While existing research has largely focused on identifying risk factors and optimizing clinical management for AIMSS,^9^ it often overlooks the complex, subjective experiences of patients navigating these symptoms in their daily lives.^10^, ^11^, ^12^ The lack of a patient-centered perspective limits our understanding of how AIMSS influences patients' overall quality of life, their coping strategies, and sustained adherence to endocrine therapy, thereby limiting the development of holistic and effective management strategies.^13^ Furthermore, most studies on AIMSS have been conducted in Western populations, which restricts the generalizability of their findings.^14^^,^^15^ Research involving Asian populations, including Chinese patients with breast cancer, remains limited. Existing study suggests that cultural beliefs^16^, ^17^, ^18^ and health care practices^19^ may influence how patients experience and report these symptoms. The emotional and psychological impacts of breast cancer, particularly anxiety, differ significantly across cultural contexts. Chinese patients typically report higher levels of anxiety than their American counterparts, highlighting the distinct psychosocial needs of this population.^20^ Additionally, research indicates that Chinese women experience lower levels of positive affect compared to American women, with a stronger correlation between low positive affect and diminished well-being.^21^ These findings suggest that emotional coping strategies and symptom management are shaped by cultural factors, which in turn influence the overall cancer treatment experience.

Therefore, this study aimed to explore the symptom experiences of musculoskeletal symptoms in Chinese patients with breast cancer receiving AI therapy, with the goal of informing the development of patient-centered, holistic symptom management strategies to improve quality of life and treatment adherence.

## Methods

### Study design

This study adopted a descriptive qualitative approach to explore the symptom experiences of musculoskeletal symptoms among patients with breast cancer undergoing AI therapy. This approach was selected to explore participants' perceptions and experiences of musculoskeletal symptoms, offering insights into how these symptoms affect their daily lives. Semi-structured telephone interviews were conducted to explore participants' symptom experiences, which facilitated a comprehensive understanding of their challenges and coping strategies. Telephone interviews were chosen for several practical reasons. First, many participants experienced physical discomfort due to musculoskeletal symptoms, limiting their mobility and making travel to the study site challenging. Second, the recruitment hospital served patients across multiple regions, creating logistical challenges for in-person interviews. Third, the follow-up care model for endocrine therapy relied on routine outpatient visits or telemedicine consultations, making telephone interviews a natural and accessible choice. Additionally, this method ensured privacy and confidentiality, encouraging participants to share their experiences openly.

### Participants

The study was conducted between June and August 2023 in the breast medical ward of a tertiary hospital in Shanghai, China. Purposive sampling, combined with maximum variance sampling, was employed to ensure diversity and representativeness in the sample. To achieve maximum variance in the sampling process, participants were selected based on the following characteristics: age (30–39, 40–49, ≥ 50 years), body mass index (BMI) (normal weight, overweight, obese), education level (high school or below, college, bachelor’s degree or above), clinical stage (I, II, III), and duration of AI therapy (< 6, ≥ 6 months). These characteristics were chosen to capture potential variability in the experience of musculoskeletal symptoms. Participants were recruited during routine outpatient visits or through follow-up calls initiated by the research team. A total of 37 women participated. Participants were selected based on the following inclusion criteria: (1) diagnosis of HR ​+ ​breast cancer;^22^ (2) clinical stage I to III, (3) undergoing AI therapy; (4) experiencing musculoskeletal symptoms; (5) ability to communicate and comprehend effectively; and (6) provision of informed consent. Exclusion criteria included: (1) cancer recurrence or metastasis; (2) diagnosis of other malignancies; (3) significant systemic diseases that could confound the assessment of musculoskeletal symptoms. In this study, systemic diseases mainly referred to musculoskeletal-related conditions such as recent fractures or orthopedic surgery, arthritis (e.g., rheumatoid arthritis), and osteoporosis diagnosed according to World Health Organization (WHO) criteria, as these conditions may independently cause or exacerbate musculoskeletal symptoms. Eligibility was confirmed by the research team through a review of patients' medical records to verify diagnosis, clinical stage, and treatment status. The presence of musculoskeletal symptoms was confirmed through patient self-report during screening.

### Data collection

**Fig. 1:**
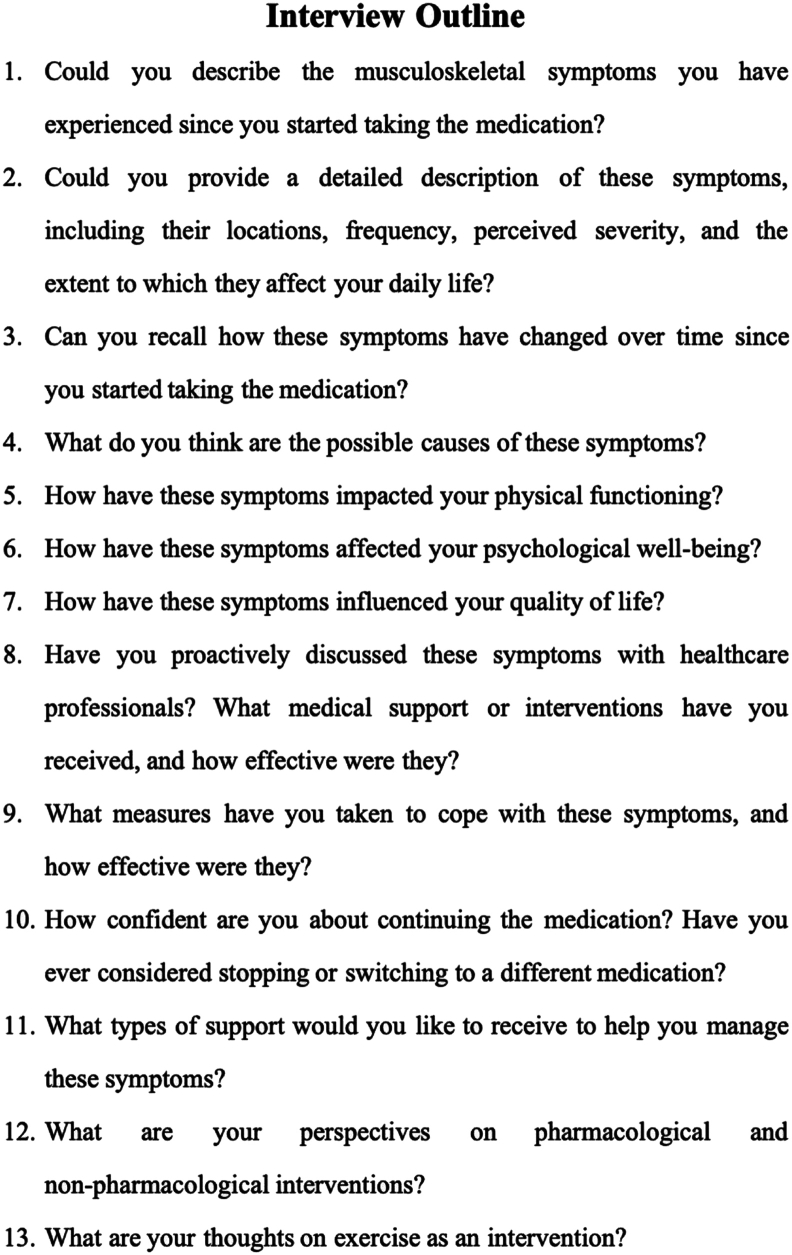
Interview outline.

This study employed semi-structured telephone interviews, with participants encouraged to select a private and distraction-free setting to ensure confidentiality and comfort. A pre-designed interview guide, developed based on the study objectives and relevant literature (Fig. 1), served as a structured framework for exploring participants' symptom experiences.

The interviews were conducted by graduate students in nursing with prior qualitative research experience. Before initiating data collection, the researchers underwent additional training to enhance their interviewing techniques, align their methods with the study objectives, and engage in reflexivity exercises to identify and mitigate potential biases.

Researchers followed a semi-structured interview guide and dynamically adjusted their questioning in response to participants' narratives. Follow-up questions clarified ambiguities, explored unexpected themes, and allowed for deeper insights into participants' experiences. Insights from earlier interviews were used to refine the interview guide for subsequent sessions, allowing researchers to explore newly emerging themes in greater depth. Each interview lasted approximately 40 minutes and continued until data saturation was achieved. Saturation was defined as the point at which no new themes or insights emerged, signifying the adequacy of data for answering the research questions.

### Data analysis

Data analysis was conducted concurrently with data collection. Audio recordings were transcribed verbatim to create a comprehensive database. Data were analyzed using thematic analysis to identify key patterns and themes. First, researchers thoroughly reviewed the transcripts to gain familiarity with participants' experiences. Meaningful data segments relevant to the research questions were identified, extracted, and coded. To maintain anonymity, data from each participant were anonymized using unique codes (e.g., P1, P2, …, P37) and imported into NVivo 14.0 software for organization and coding. After initial coding, the researchers grouped similar codes into broader themes through an iterative process of thematic analysis, refining the categories to develop a comprehensive understanding of the data. To ensure analytical rigor, multiple strategies were employed to validate the themes. First, a third researcher independently audited the codes and themes to confirm consistency and identify any discrepancies. Second, the preliminary findings were reviewed by field experts, who provided constructive feedback to refine the analysis. Third, selected participants were invited to verify the themes, ensuring they accurately reflected their symptom experiences. Any discrepancies in coding or interpretation were addressed through iterative team discussions, which were guided by reflexivity to minimize biases and maintain analytical neutrality. The finalized themes were synthesized into a coherent narrative, enriched with direct quotations from participants to illustrate key aspects of their symptom experiences.

### Quality control

To ensure scientific rigor, all researchers received training in qualitative research methods before conducting the interviews. A maximum variance sampling strategy was implemented to enhance representativeness, taking into account demographic, disease, and treatment-related factors. During interviews, researchers ensured neutrality by clarifying ambiguities, using neutral language, and adapting questioning techniques to participants' responses. The interview recordings were independently transcribed by two researchers and the transcripts were cross-checked to minimize bias and ensure accuracy. In preparing this study, we referenced the SRQR (Standards for Reporting Qualitative Research) guidelines to ensure transparency and rigor in our reporting.

## Results

### Characteristics of participants

The study included 37 patients with a mean age of 51.51 ​± ​9.13 years and a mean BMI of 22.60 ​± ​2.34 kg/m^2^. The majority of participants were married (*n* ​= ​33, 89.2%), had a college education or higher (*n* ​= ​22, 59.5%), and were employed (*n* ​= ​19, 51.4%). Clinical stages were evenly distributed across stage I (*n* ​= ​9, 24.3%), stage II (*n* ​= ​23, 62.2%), and stage III (*n* ​= ​5, 13.5%). Exemestane was the most commonly prescribed medication (*n* ​= ​20, 54.1%), followed by letrozole (*n* ​= ​14, 37.8%) and anastrozole (*n* ​= ​3, 8.1%). More than half of the participants (*n* ​= ​25, 67.6%) had been undergoing AI therapy for over one year, while 12 participants (32.4%) had been on therapy for less than one year. Additional details regarding participant characteristics are presented in Table 1.

**Table 1:** Characteristics of participants.

No.	Age (years)	Body mass index (kg/m^2^)	Education level	Employment status	Marital status	Clinical stage	Medication	Therapy time
1	42	20.3	Junior high school or below	Employed	Divorced	Ⅱ	Exemestane	≤ 1 year
2	44	24.2	College or above	Employed	Single	Ⅰ	Exemestane	> 1 year
3	51	23.1	College or above	Employed	Single	Ⅱ	Exemestane	> 1 year
4	60	22.4	College or above	Unemployed	Married	Ⅱ	Letrozole	≤ 1 year
5	38	23.2	College or above	Employed	Married	Ⅱ	Exemestane	> 1 year
6	43	18.6	College or above	Employed	Married	Ⅰ	Letrozole	≤ 1 year
7	42	21.2	College or above	Unemployed	Married	Ⅲ	Exemestane	> 1 year
8	43	24.2	College or above	Employed	Married	Ⅱ	Exemestane	≤ 1 year
9	64	29.7	College or above	Unemployed	Married	Ⅱ	Letrozole	> 1 year
10	53	23.1	College or above	Employed	Married	Ⅱ	Exemestane	≤ 1 year
11	63	23.5	High school	Unemployed	Married	Ⅱ	Letrozole	> 1 year
12	38	22.1	College or above	Employed	Married	Ⅰ	Exemestane	> 1 year
13	49	20.3	Junior high school or below	Employed	Married	Ⅱ	Letrozole	> 1 year
14	54	19.8	College or above	Unemployed	Married	Ⅲ	Exemestane	> 1 year
15	51	20.0	College or above	Unemployed	Married	Ⅱ	Letrozole	> 1 year
16	46	23.7	College or above	Employed	Married	Ⅰ	Letrozole	≤ 1 year
17	64	23.9	High school	Unemployed	Married	Ⅱ	Letrozole	> 1 year
18	43	22.0	College or above	Employed	Married	Ⅱ	Exemestane	> 1 year
19	61	22.0	College or above	Unemployed	Married	Ⅱ	Letrozole	≤ 1 year
20	43	23.5	Junior high school or below	Employed	Divorced	Ⅰ	Letrozole	> 1 year
21	66	21.0	High school	Unemployed	Married	Ⅱ	Anastrozole	> 1 year
22	44	27.9	College or above	Employed	Married	Ⅰ	Anastrozole	≤ 1 year
23	47	26.6	High school	Unemployed	Married	Ⅱ	Exemestane	> 1 year
24	73	22.7	Junior high school or below	Unemployed	Married	Ⅲ	Letrozole	> 1 year
25	67	23.0	High school	Unemployed	Married	Ⅱ	Exemestane	≤ 1 year
26	43	20.8	College or above	Employed	Married	Ⅰ	Exemestane	> 1 year
27	56	18.7	Junior high school or below	Unemployed	Married	Ⅱ	Exemestane	> 1 year
28	62	23.8	High school	Unemployed	Married	Ⅱ	Exemestane	≤ 1 year
29	54	23.9	College or above	Unemployed	Married	Ⅰ	Exemestane	> 1 year
30	46	21.3	College or above	Employed	Married	Ⅱ	Exemestane	> 1 year
31	53	24.1	College or above	Employed	Married	Ⅱ	Exemestane	> 1 year
32	47	21.4	High school	Unemployed	Married	Ⅱ	Exemestane	> 1 year
33	52	23.5	High school	Unemployed	Married	Ⅱ	Letrozole	> 1 year
34	52	22.9	College or above	Employed	Married	Ⅰ	Letrozole	> 1 year
35	52	20.0	College or above	Employed	Married	Ⅱ	Letrozole	> 1 year
36	60	24.0	Junior high school or below	Unemployed	Married	Ⅲ	Exemestane	≤ 1 year
37	40	19.8	Junior high school or below	Employed	Married	Ⅲ	Anastrozole	≤ 1 year

Participant-reported experiences of musculoskeletal symptoms are summarized in Table 2.

**Table 2:** Participant experiences of musculoskeletal symptoms in patients with breast cancer undergoing aromatase inhibitor therapy.

Theme	Sub-themes	Codes	Representative quotes
Symptom experience and perception	Persistent pain and joint stiffness	Persistent pain	“If I take the medication, my index fingers hurt a lot in the morning. When I wake up, my hands are completely bent, and it hurts so much that I can’t straighten them.” (P23)
		Joint stiffness	“Morning stiffness is the most noticeable. Then, during the day, if I rest and sit still for a while, it’s a bit difficult to move suddenly when I get up.” (P35)
		Fatigue and weakness	“Whenever I climb stairs, I definitely hold onto the handrail. Otherwise, even if I’m walking fine, my knee might suddenly give out, and I’ll kneel down unexpectedly.” (P7)
		Secondary symptoms	“Sometimes I also experience dizziness, muscle cramps, and nausea, which makes daily life even more uncomfortable.”
	Cyclical and fluctuating symptom trajectories	Severity of morning stiffness	“Stiffness occurs every day, especially when getting up in the morning. It’s particularly severe in the morning.” (P34)
		Temporary relief and recurrence	“It’s not continuous pain. For example, after sitting for a long time and then standing up, my bones feel very stiff, and my legs become extremely stiff. I Need to move a bit before I can do other things.” (P34)
		Symptom change over time	“After taking the medication for about three months, the stiffness became noticeable. It wasn’t until around 10 months later that it eased slightly.” (P2)
		Symptom worsening	“At the beginning, during the first one to two months of taking the medication, it wasn’t the worst. The morning stiffness would gradually get better after waking up. But later on, it became increasingly severe. My fingers couldn’t even function.” (P29)
The dual dilemma of psychological and social functioning	Navigating emotional and social impacts of functional limitations	Emotional frustration and social withdrawal	“How can I be happy when I can’t even walk properly? Climbing stairs every day has become my biggest exercise, and I don’t dare to interact with others. I Basically just stay at home.” (P33)
		Inability to perform household tasks	“It’s the pain in my shoulder. Sometimes it’s difficult to hold a child or do certain things.” (P19)
		Communication challenges in family roles	“The pain still bothers my life, including going out to do things—it’s still affected … My child isn’t particularly understanding, which causes a lot of frustration. Plus, my husband doesn’t seem to understand me very well either.” (P16)
		Impact on family relationships	“When I’m not in a good mood, it might affect my family. For example, when I’m irritable, I vent my frustrations on them.” (P34)
	From uncertainty to balance	Negative emotions and uncertainty	“It has a big impact on my life because my feet can’t stand properly; it always hurts and keeps reminding me of the discomfort. Sometimes I feel irritable and depressed, and I can’t control my emotions very well.” (P16)
		Financial pressures exacerbating stress	“This medication is expensive, and we’re paying out of pocket. I Frequently travel back and forth between Shanghai and Chongqing, which adds a lot of financial pressure.” (P36)
		Family support as emotional and practical aid	“I definitely can’t do without my family’s support and understanding.” (P32) ​ “My family is great; they’re always supportive and encouraging. I Also participate in social activities outside.” (P25)
		Symptom understanding fostering control	“Gaining a clearer understanding of my symptoms and their progression over time really helped me feel more in control of the situation.” (participant 32)
Coping mechanisms and support systems	Personalized coping and integrative practices	Exercise for symptom relief	“If I’m lazy and don’t move, I feel very stiff. But if I exercise, do swimming or yoga, I feel like my body loosens up.” (P18)
		Other non-pharmacological interventions	“Now I go for acupuncture and massage every week, and I also do moxibustion on my own. I Think it’s helpful; at least for a few days after the sessions, I feel relatively comfortable.” (P15)
		Pharmacological interventions	“I’ve been taking calcium tablets recently. I Used to feel a bit uncomfortable going downstairs, but after taking them for about half a month, it seems like my knees are getting better.” (P11)
		Integrative approaches (combined strategies)	“Combining Chinese medicine, calcium supplementation, and dietary control seems to have a better effect when done together.” (P20)
	The synergistic role of support systems	Support from health care professionals	“Whenever I feel unwell, I communicate with the doctors during my follow-ups.” (P31)
		Family support for physical and emotional relief	“Upon confiding in my family, they habitually assume responsibility for certain tasks, bestowing profound emotional sustenance that imbues me with a sense of empowerment.” (P31)
		Community networks for dual support	“I learned some health exercises from the older women in the community, such as Baduanjin and Tai Chi. I Would join the uncles and aunts there for activities.” (P3)
Informational challenges and support strategies	Barriers to accessing reliable information	Limited communication with health care providers	“Your hospital is too far away. Now I go there once a year for a check-up … The time to communicate with doctors is relatively limited because doctors are also very busy.” (P34)
		Geographical barriers	“Because I live far away, it’s not realistic for me to visit your hospital every time.” (P8)
	Turning to informal sources	Peer-established networks	“We also have a patient group, and sometimes we see people asking each other questions there. It’s quite helpful.” (P18)
		Online platforms and family advice	“The support you can provide for us is to tell us what areas to pay attention to in daily life, like what to watch for in terms of nutrition and diet.” (P1)
	Demand for tailored and accessible information	Need for professional guidance	“I really want clear advice from the doctors about what exercises I should do to relieve my stiffness and what kind of food is good for my joints.” (P1)
		Preference for remote and accessible platforms	“I’m hoping for remote support to help manage these symptoms, how to relieve them, and how to prevent them.” (P8)
		Desire for a combined approach	“Combining professional guidance with online platforms would help us better manage symptoms and access information conveniently.” (P8)

### Theme 1 Symptom experience and perception

#### Uncomfortable pain and stiffness

Pain was the most commonly reported symptom, frequently described as persistent and disruptive. Participants emphasized its impact on daily activities, particularly in the hands and knees. One participant (P23) shared, *“If I take the medication, my index fingers hurt a lot in the morning. When I wake up, my hands are completely bent, and it hurts so much that I can’t straighten them”*. Joint stiffness was another frequently mentioned symptom, especially severe in the mornings. Participants described a “locking” sensation in their joints, which hindered essential tasks such as opening doors. One participant (P35) reflected, *“Morning stiffness is the most noticeable. Then, during the day, if I rest and sit still for a while, it’s a bit difficult to move suddenly when I get up”*. Another participant (P3) added, *“When my hands are stiff, I have to move slower when getting on the bed. Going down also hurts a bit; when my legs are stiff, getting out of the car and things like that are a bit difficult”*. Although less commonly mentioned than pain and stiffness, fatigue was described as persistent weakness exacerbating joint discomfort. Participants noted that fatigue intensified challenges in managing daily activities. One participant (P7) shared, *“Whenever I climb stairs, I definitely hold onto the handrail. Otherwise, even if I’m walking fine, my knee might suddenly give out, and I’ll kneel down unexpectedly”*. Some participants also reported secondary symptoms such as dizziness, muscle cramps, and nausea, which added to the discomfort of daily life.

#### Persistent and fluctuating symptom trajectories

Participants characterized their musculoskeletal symptoms as cyclical and condition-dependent, emphasizing noticeable daily fluctuations. Morning stiffness, particularly in the wrists and finger joints, was frequently reported as a significant barrier to starting the day. One participant (P17) explained, *“Stiffness occurs every day, especially when getting up in the morning. It’s particularly severe in the morning”*. However, relief from morning stiffness was typically temporary, as symptoms often recurred later in the day, further disrupting daily routines. Participant (P34) remarked, *“It’s not continuous pain. For example, after sitting for a long time and then standing up, my bones feel very stiff, and my legs become extremely stiff. I need to move a bit before I can do other things*”.

The progression of symptoms over time varied widely among participants. Some described gradual improvement or stabilization of early-stage symptoms, such as reduced morning stiffness, after continued treatment. Participant (P2) noted, *“After taking the medication for about three months, the stiffness became noticeable. It wasn’t until around ten months later that it eased slightly”*. In contrast, others reported persistent or worsening symptoms, including increased joint pain and the emergence of additional complications, such as muscle weakness. As Participant (P29) shared, *“At the beginning, during the first one to two months of taking the medication, it wasn’t the worst. The morning stiffness would gradually get better after waking up. But later on, it became increasingly severe. My fingers couldn’t even function. At first, there weren’t these symptoms, but they gradually worsened over time”*. In several cases, participants delayed seeking professional advice or attributed initial symptoms to “normal aging,” a response pattern consistent with healthcare-seeking behaviors observed in Chinese contexts, where medical attention is often sought only when symptoms substantially impair daily functioning.

### Theme 2 The dual dilemma of psychological and social functioning

#### Navigating emotional and social impacts of functional limitations

Musculoskeletal symptoms had profound psychosocial and functional consequences, significantly affecting participants' emotional well-being, social interactions, and ability to fulfill daily responsibilities. Persistent pain and stiffness frequently led to frustration, feelings of inadequacy, and social withdrawal. Continuous discomfort often made it difficult to maintain emotional resilience, resulting in isolation from society. One participant (P33) reflected, “How can I be happy when I can’t even walk properly? Climbing stairs every day has become my biggest exercise, and I don’t dare to interact with others. I basically just stay at home.” In the Chinese cultural context, women often bear primary responsibility for household and caregiving duties. When musculoskeletal symptoms hindered their ability to perform these roles, participants reported heightened feelings of inadequacy and family tension, reflecting the strong cultural association between fulfilling domestic responsibilities and self-worth. This inability to meet expected household and caregiving responsibilities not only created emotional strain within familial relationships but also led to pronounced role conflicts, further undermining participants' sense of identity.

The persistent nature of pain and stiffness limited participants' ability to perform household tasks and, in turn, strained family relationships. For instance, one participant (P19) noted, “It’s the pain in my shoulder. Sometimes it’s difficult to hold a child or do certain things.” In addition, communication challenges within family relationships emerged as another source of stress. One participant (P16) explained, “The pain still bothers my life, including going out to do things—it’s still affected … My child isn’t particularly understanding, which causes a lot of frustration. Plus, my husband doesn’t seem to understand me very well either.” Another participant (P34) remarked, “When I’m not in a good mood, it might affect my family. For example, when I’m irritable, I vent my frustrations on them.” These experiences illustrate how functional limitations can disrupt family dynamics and precipitate conflicts in fulfilling expected roles, thereby intensifying the emotional burden on patients.

#### From uncertainty to balance

At the onset of treatment, many participants reported experiencing negative emotions, such as anxiety, distress, and depressive symptoms, which they attributed to the physical discomfort and uncertainty associated with musculoskeletal symptoms. For example, participant (P16) shared, “It has a big impact on my life because my feet can’t stand properly; it always hurts and keeps reminding me of the discomfort. Sometimes I feel irritable and depressed, and I can’t control my emotions very well.” As symptoms persisted, the heightened sense of uncertainty contributed to further emotional strain. However, over time, participants reported gaining a clearer understanding of their symptoms and their progression, which alleviated the uncertainty and fostered a greater sense of control. As participant (P15) explained, “At first, I was scared and anxious, wondering if things would improve. After six months of treatment, I was already feeling so uncomfortable that I worried the medication, which lasts 10 years, might make my condition worse. I feared bone loss and osteoporosis. But as I started feeling better, I stopped worrying, realizing that if any bone issues arise, I can just face them and deal with it.” This shift in mindset—from fear to acceptance—was central to participants' transition toward a more balanced emotional state. Family support emerged as a critical factor in alleviating emotional distress and reinforcing the sense of balance. Many participants emphasized the importance of a supportive family network in coping with ongoing challenges. Participant (P32) remarked, “I definitely can’t do without my family’s support and understanding”, and another participant (P25) added, “My family is great; they’re always supportive and encouraging. I also participate in social activities outside.” Such reliance on family aligns with the collectivist values in Chinese culture, where emotional coping and decision-making are often embedded within close family networks rather than sought from external professional sources. These supportive dynamics, coupled with an increasing understanding of their health, allowed participants to navigate the emotional turbulence of treatment and gradually regain a sense of balance.

### Theme 3 Coping mechanisms and support systems

#### Personalized coping and integrative practices: a multidimensional exploration of musculoskeletal symptom management

Participants widely reported utilizing non-pharmacological interventions to manage musculoskeletal symptoms, particularly joint pain and stiffness, and improve their quality of life. Exercise was the most commonly chosen intervention, frequently cited for its ability to reduce stiffness, enhance joint mobility, and alleviate discomfort. Common activities included walking, yoga, swimming, aerobics, and traditional practices like Baduanjin. One participant (P18) shared, *“If I’m lazy and don’t move, I feel very stiff. But if I exercise, do swimming or yoga, I feel like my body loosens up”*. Patients generally preferred exercises that minimized joint stress and aligned with their physical abilities. Beyond exercise, participants adopted other non-pharmacological interventions targeting localized discomfort, such as physiotherapy, acupuncture, sunbathing, and topical Chinese medicine. For instance, one participant (P12) noted, *“Now I go for acupuncture and massage every week, and I also do moxibustion on my own. I think it’s helpful; at least for a few days after the sessions, I feel relatively comfortable”*. The use of Tai Chi, Baduanjin, acupuncture, and moxibustion reflects Chinese cultural health beliefs emphasizing balance, energy flow, and holistic well-being. These practices are embedded in daily life and viewed as safe, natural options, aligning with patients' preference to limit long-term medication use.

However, several participants acknowledged that exercise alone was insufficient to address all symptoms comprehensively. As Participant (P35) explained, “It must be treated with medication because it’s a side effect of endocrine therapy. Natural interventions alone aren’t sufficient; medical intervention is necessary.” Another participant (P12) similarly remarked, “I don’t believe this medication-related problem can be solved just by moving around; you still need to take medicine to deal with the side effects.” These perspectives reflected a broader sentiment among participants that pharmacological interventions were essential, particularly for alleviating persistent joint pain and preventing long-term complications. Calcium supplementation was the most frequently reported option, with 21 out of 38 participants noting improvements in joint discomfort and mobility. For example, Participant (P11) shared, “I’ve been taking calcium tablets recently. I used to feel a bit uncomfortable going downstairs, but after taking them for about half a month, it seems like my knees are getting better.” In addition to calcium supplements, a smaller group used bisphosphonate therapy, primarily to address significant bone loss. Some participants chose to integrate traditional Chinese medicine (TCM) into their management plans, especially to address joint pain and sleep disturbances. Participant (P15) recalled, “Initially, I was not sleeping well, so I took some Chinese medicine. After a while, I adjusted, and my sleep improved.” The integration of TCM with conventional pharmacological treatments reflects the enduring role of culturally rooted health practices in symptom management, even within modern oncology care in China.

Several participants emphasized the benefits of an integrative approach to managing joint pain and stiffness, combining pharmacological treatments with non-pharmacological interventions for improved symptom relief. This approach included incorporating exercise, dietary adjustments, and alternative therapies into their routines. One participant (P20) expressed, *“Combining Chinese medicine, calcium supplementation, and dietary control seems to have a better effect when done together”*. This integrative approach aligns with the Chinese holistic health paradigm, which seeks to harmonize the body by combining modern biomedicine with traditional health practices.

#### The synergistic role of support systems

Participants emphasized the importance of multiple support systems in managing the physical and emotional challenges associated with musculoskeletal symptoms, particularly joint pain and stiffness. Participants frequently described health care professionals as key sources of support, offering practical strategies for addressing joint-related discomfort and managing symptoms. As one participant (P31) shared, *“Whenever I feel unwell, I communicate with the doctors during my follow-ups”*. Family members also played a pivotal role in alleviating the physical and emotional toll of symptoms. Participants often relied on spouses or close relatives for assistance with physically demanding tasks, such as household chores, which helped reduce physical strain. Participant (P31) further explained, *“Upon confiding in my family, they habitually assume responsibility for certain tasks, bestowing profound emotional sustenance that imbues me with a sense of empowerment”*. In addition, community networks provided additional layers of support. Group activities such as Tai Chi and Baduanjin were particularly valued for their dual benefits of therapeutic exercise and social interaction. Participant (P3) noted, *“I learned some health exercises from the older women in the community, such as Baduanjin and Tai Chi. I would join the uncles and aunts there for activities. Before I got sick, I used to love exercising and even played ball games with them when I was younger”*. Reliance on family members and participation in community-based exercise groups reflect the collectivist orientation of Chinese society, where caregiving is shared within households and social networks actively support symptom management by providing both therapeutic benefits and emotional support, helping to mitigate the isolation associated with chronic musculoskeletal symptoms.

### Theme 4 Informational challenges and support strategies in the management of musculoskeletal symptoms

Many participants reported considerable challenges in obtaining adequate information to effectively manage their musculoskeletal symptoms, particularly joint pain and stiffness. Limited communication with health care providers was a recurring issue, often caused by the disproportionate ratio of providers to patients and geographical barriers. Participant (P34) explained, *“Your hospital is too far away. Now I go there once a year for a check-up. If the check-up shows no major issues, I just come back. The time to communicate with doctors is relatively limited because doctors are also very busy. If I feel there’s no significant issue, I won’t bother to see them”*. This pattern reflects the highly centralized nature of cancer care in China, where specialized oncology services are concentrated in major urban hospitals. As a result, patients living in rural or remote areas often face substantial travel burdens and limited contact time with providers, shaping their reliance on infrequent, high-intensity consultations. This lack of direct interaction left many patients feeling under-informed about symptom management strategies, such as exercises for stiffness relief or medication use.

To bridge these gaps, many participants turned to informal sources, including peer-established networks, online platforms, and advice from family or friends with similar experiences. The preference for peer-to-peer advice and community networks is reinforced by the collectivist orientation of Chinese society, with health-related decision-making drawing on shared experiences within social circles. As participant (P18) noted, *“We also have a patient group, and sometimes we see people asking each other questions there. It’s quite helpful”*. In China, online patient groups hosted on platforms such as WeChat serve as both social and informational hubs, offering easily accessible exchanges that complement—but cannot replace—formal medical advice. While these alternative pathways provided some degree of support, they often lacked the reliability of professional guidance. Over half of the participants (63.2%, or 24 out of 38) expressed the pressing need for targeted informational support related to their bone and joint symptoms. Core areas of demand included guidance on physical activity and dietary adjustments, medication regimens, and understanding the progression of musculoskeletal conditions. Participant (P1) stated, *“The support you can provide for us is to tell us what areas to pay attention to in daily life, like what to watch for in terms of nutrition and diet”*. Similarly, Participant (P8) highlighted the need for remote resources, explaining, *“Because I live far away, it’s not realistic for me to visit your hospital every time. So, I’m hoping for remote support to help manage these symptoms, how to relieve them, and how to prevent them”*. Participants consistently emphasized the importance of combining professional guidance with accessible and immediate platforms, such as online resources, to meet their symptom management needs effectively. This dual approach underscored their desire for both reliability and convenience in accessing information critical to managing their musculoskeletal symptoms. Such requests for remote support highlight the growing acceptance of telemedicine and digital health solutions in China, where mobile health platforms are increasingly viewed as viable supplements to traditional hospital-based care.

## Discussion

### Navigating musculoskeletal symptoms: patient perception and behavioral adjustments

This study revealed the complex nature of musculoskeletal symptoms experienced by patients with breast cancer undergoing AI therapy, particularly the uncertainty surrounding symptom recurrence and timing, which significantly limited their ability to initiate daily tasks. Drawing on the symptom experience model (SEM),^23^ the findings highlighted a dynamic relationship between symptom perception, behavioral adaptations, and their subsequent impact on daily functioning. Notably, cultural norms in China shaped patients' interpretation of pain. Many participants perceived musculoskeletal pain as a bearable hardship, rooted in traditional values of endurance and stoicism.^24^ This cultural framing led some patients to delay seeking medical help. Patients' perceptions of symptoms, such as the severity and timing of morning stiffness, directly influenced their behavioral adaptations, including adjustments to the pace and sequence of daily activities. These adaptive strategies reflected patients' resilience and self-efficacy in managing symptoms,^25^^,^^26^ contributing to a partial—though not complete—mitigation of disruptions in mobility and productivity. The cyclical and fluctuating nature of musculoskeletal symptoms underscores the dynamic interplay between symptom perception and behavioral adjustments.^27^ These findings emphasize the need to incorporate the multidimensional characteristics of symptoms into nursing interventions to better support patients' self-management and enhance their behavioral adaptations.

### The interplay of psychological and social functioning in response to musculoskeletal symptoms

This study highlights the significant psychological and social challenges faced by patients with breast cancer undergoing AI therapy due to musculoskeletal symptoms. Persistent pain, stiffness, and fatigue not only imposed significant emotional burdens but also disrupted participants' ability to maintain familial and social roles.^28^ Symptoms such as morning stiffness and fatigue interfered with daily routines, causing frustration and a reduced sense of social connectedness.^29^ These disruptions often created a reinforcing cycle in which emotional distress exacerbated difficulties in managing symptoms, further limiting patients' capacity to engage in meaningful social interactions.

The dynamic relationship between psychological and social functioning emerged as a central theme in participants' experiences, driven by the persistent impact of musculoskeletal symptoms. Symptoms such as joint pain, stiffness, and fatigue disrupted participants' ability to fulfill familial and social roles, intensifying emotional strain and fostering feelings of isolation. In the context of Chinese patients, cultural norms such as the emphasis on family roles and social harmony significantly influence how patients perceive and respond to these symptoms.^30^ Many Chinese patients feel a heightened sense of responsibility toward their families, which may make them reluctant to openly report symptoms or seek external social support. This responsibility is particularly pronounced among women, as traditional gender roles in China often position them as primary caregivers within households. Studies indicate that Chinese female patients frequently prioritize familial duties over self-care, viewing symptom reporting as a potential disruption to family harmony. This prioritization can exacerbate psychological distress, as unmanaged symptoms conflict with their perceived obligations.^31^^,^^32^ These cultural expectations, coupled with emotional distress, create a cycle that complicates seeking help, further reinforcing isolation and psychological burden.^33^ However, despite these cultural norms, robust familial support and structured daily routines still help patients mitigate the impact of musculoskeletal symptoms, enabling them to stabilize emotionally and re-engage with social roles.^34^ This illustrates how cultural values, while fostering resilience, can also contribute to feelings of isolation. Furthermore, social resources not only facilitated the navigation of daily challenges but also acted as a critical emotional buffer, alleviating psychological strain and fostering a greater sense of resilience.^35^, ^36^, ^37^ These findings suggest that effective strategies for managing musculoskeletal symptoms should focus on strengthening both familial and external social support networks^38^ and equipping patients with tailored coping mechanisms to address their psychological and social needs effectively.

### Personalized coping strategies and the potential of integrated practices

This study highlights the variety of personalized strategies that patients with breast cancer use to manage musculoskeletal symptoms associated with AI therapy. Participants expressed diverse preferences, including both non-pharmacological and pharmacological interventions.

Participants consistently reported utilizing non-pharmacological interventions to manage musculoskeletal symptoms, particularly joint pain and stiffness, while also aiming to improve their overall quality of life. Exercise emerged as the most frequently selected intervention, with participants noting its effectiveness in reducing stiffness, enhancing joint mobility, and alleviating discomfort. Common activities included walking, yoga, swimming, aerobics, and traditional practices such as Baduanjin. As one participant (P18) shared, “If I’m lazy and don’t move, I feel very stiff. But if I exercise, do swimming or yoga, I feel like my body loosens up.” Patients generally preferred exercises that minimized joint stress and aligned with their physical capabilities. In addition to exercise, participants reported that physiotherapy sessions provided targeted relief for joint pain. Some participants also turned to traditional Chinese medicine practices, such as acupuncture and moxibustion, which were valued for their ability to promote relaxation and improve overall well-being. These non-pharmacological interventions, often serving as primary management strategies, provide symptom relief while avoiding the adverse effects associated with medications.

However, several participants acknowledged that exercise alone was insufficient to address all symptoms comprehensively. Participant (P35) stated,“It must be treated with medication because it’s a side effect of endocrine therapy. Natural interventions alone aren’t sufficient; medical intervention is necessary.” Pharmacological interventions were considered essential by many participants, particularly for alleviating persistent joint pain and preventing long-term complications. Calcium supplementation was the most frequently mentioned treatment, with 21 out of 38 participants reporting improvements in joint discomfort and mobility. As one participant (P11) explained,” I’ve been taking calcium tablets recently. I used to feel a bit uncomfortable going downstairs, but after taking them for about half a month, it seems like my knees are getting better.” In addition to calcium supplements, bisphosphonate therapy was used by a smaller subset of participants, primarily to address severe osteoporosis. Traditional Chinese medicine was another pharmacological option favored by some participants, particularly for managing symptoms such as joint pain and sleep disturbances. For example, one participant (P15) noted, “Initially, I was not sleeping well, so I took some Chinese medicine. After a while, I adjusted, and my sleep improved.”

Several participants emphasized the benefits of an integrative approach to managing joint pain and stiffness, combining pharmacological treatments with non-pharmacological interventions for improved symptom relief. This approach often involved incorporating exercise, dietary adjustments, and alternative therapies into their daily routines. One participant (P20) expressed, “Combining Chinese medicine, calcium supplementation, and dietary control seems to have a better effect when done together.”

Notably, a predominant trend emerged in favor of combining pharmacological and non-pharmacological interventions,^39^^,^^40^ reflecting a holistic approach that leverages the complementary benefits of these interventions. The preference for integrated management strategies underscores potential gaps in conventional care practices, which often rely on standardized regimens and fail to address the complex, individualized needs of patients with breast cancer experiencing musculoskeletal symptoms.^41^ Incorporating patient-preferred approaches, such as combining pharmacological and non-pharmacological interventions, into routine care may better address the dual challenges of symptom relief and maintaining daily functioning. Aligning clinical symptom management with patient preferences has the potential to enhance treatment adherence and improve overall quality of life.^42^ Future efforts should focus on refining care frameworks to integrate these diverse strategies, ensuring a more holistic and responsive approach to symptom management in this population.

### Optimizing information support for musculoskeletal symptom management: bridging accessibility and precision

The findings highlight patients with breast cancer face significant challenges in accessing reliable, condition-specific information to manage musculoskeletal symptoms from AI therapy. Many participants reported difficulties accessing timely and authoritative guidance on symptom management and specific interventions.^39^^,^^43^ In China, health care system constraints, including overburdened public hospitals and limited individualized patient education, further exacerbate these challenges. As a result, many patients rely on informal sources such as peer networks and online platforms.^44^^,^^45^ However, the reliability of these sources varies considerably, often leading to misinformation and confusion. While these sources provided some support, they frequently lacked the accuracy required for effective symptom management.^46^ Culturally, some Chinese patients integrate family and peer recommendations alongside medical advice, balancing multiple perspectives in decision-making. While social networks provide emotional support, they may also introduce conflicting information, complicating symptom management. Consequently, some patients adopt mixed approaches that incorporate both medical guidance and informal recommendations.^47^ This variability in information quality highlights the need for tailored, evidence-based resources and enhanced digital health literacy. Addressing these needs requires a dual-pronged approach: integrating professional expertise into user-friendly resources and improving the immediacy and accessibility of these tools.^48^ Rather than relying on individual health care providers to engage with social media, public health authorities and medical institutions should lead efforts to establish verified, authoritative medical sources. Additionally, strengthening digital health literacy initiatives can help patients critically evaluate online content and discern reliable sources from misinformation. For example, enhancing remote consultation services could provide timely access to professional guidance, while optimizing digital platforms might deliver evidence-based resources tailored to individual needs more effectively.^49^ By aligning informational support with the specific challenges of musculoskeletal symptom management, health care systems can better support patient self-management and ultimately improve quality of life.^50^^,^^51^

### Study limitations and future directions

Despite the valuable insights generated by this study, several limitations must be acknowledged. First, the participants were drawn from a single tertiary hospital in Shanghai, which may limit the generalizability of the findings to populations with different cultural or health care contexts. Second, the majority of participants had received higher education, which may have influenced their health literacy, access to resources, and ability to manage symptoms. This could result in findings that are fewer representatives of individuals with lower educational backgrounds, who may face greater challenges in recognizing, communicating, and managing musculoskeletal symptoms. Third, while semi-structured telephone interviews provided convenience, they may have restricted the ability to capture non-verbal cues, which could offer deeper insights into participants' experiences. Finally, although self-reported data are inherently subject to recall bias or social desirability effects, these limitations were mitigated through participant verification and thematic saturation, enhancing the credibility and reliability of the findings.

Future research should address these limitations by recruiting a more diverse and geographically representative sample to improve the generalizability of findings. Incorporating longitudinal designs would allow for tracking changes in symptom management over time, while clinical trials could evaluate the effectiveness of integrated interventions. Furthermore, incorporating objective measures of symptom burden and functional outcomes could enhance the applicability and translational value of the findings.

## Conclusions

This study offers an in-depth exploration of the musculoskeletal symptom experiences of patients with breast cancer undergoing AI therapy, revealing the multifaceted challenges posed by these symptoms. Joint pain, stiffness, and fatigue emerged as pervasive and cyclical issues that disrupted patients' daily routines, imposed significant emotional burdens, and impaired their social functioning. Although patients displayed resilience by adopting adaptive coping strategies, their efforts were often constrained by limited access to reliable information and professional support.

By situating these experiences within the context of patients' daily lives, this study deepens the understanding of how patients with breast cancer perceive, navigate, and adapt to the complexities of musculoskeletal symptoms. The findings underscore the critical need for patient-centered care approaches that integrate personalized symptom management strategies and improve access to reliable, evidence-based resources. These insights provide a foundation for refining clinical practices and developing targeted interventions to enhance both symptom control and quality of life for this population.

## CRediT authorship contribution statement

**Yuling CAO, Feng JING** and **Yan HU:** Conceptualization; **Yuling CAO, Feng JING, Maoting TIAN, Lingyun JIANG, Jiajia Qiu, Lichen Tang,** and **Yan HU:** Methodology; **Yuling CAO** and **Feng JING:** Writing – original draft; **Yuling CAO, Feng JING,** and **Yan HU:** Writing – review and editing. All authors had full access to all the materials and transcripts in the study, and the corresponding author had final responsibility for the decision to submit for publication.

## Ethics statement

The study received ethical approval from the Ethics Committee of Fudan University Shanghai Cancer Center (Approval No. 2304273-17). The study was conducted in accordance with the Declaration of Helsinki. Written informed consent was obtained from all participants prior to data collection. Confidentiality and anonymity were strictly maintained throughout the study.

## Data availability statement

The data that support the findings of this study are managed by the first author, YC, and are available upon reasonable request via the corresponding author, YH.

## Declaration of generative AI and AI-assisted technologies in the writing process

During the preparation of this work, the authors used ChatGPT (OpenAI) solely for language refinement to improve clarity and readability. The authors reviewed and edited all content generated by this tool and take full responsibility for the final version of the manuscript.

## Funding

This research is supported by two research grants: (1) 2023 JBI Bright Future Grant; (2) 2023 National Natural Science Foundation of China (NSFC), Grant No. 2023-82272922. The funders had no role in considering the study design or in the collection, analysis, interpretation of data, writing of the report, or decision to submit the article for publication.

## Declaration of competing interest

All authors declare no conflicts of interest. Professor Yan Hu, the corresponding author, serves on the editorial board of the *Asia–Pacific Journal of Oncology Nursing*. The article underwent standard review procedures of the journal, with peer review conducted independently of Professor Hu and their research groups.
